# Genome-Wide Identification, Characterization, and Expression Analysis of Trehalose Metabolism Genes in Tea Plant (*Camellia sinensis*) Reveals Their Roles in Response to Heat Stress

**DOI:** 10.3390/plants14213309

**Published:** 2025-10-29

**Authors:** Shizhong Zheng, Xiaohui Chen, Ziwei Zhou, Rongzhao Lin, Huangxin Jiang, Liyi Xu, Jingjing Su

**Affiliations:** 1 College of Biological Science and Engineering, Ningde Normal University, Ningde 352100, China; zhengshizhong@126.com (S.Z.); zwchow92@126.com (Z.Z.); xuebizhenyin@gmail.com (R.L.); 13616095691@163.com (H.J.); xuliyi1990@outlook.com (L.X.); sujingjing0304@outlook.com (J.S.); 2 College of Life Sciences, Fujian Normal University, Fuzhou 350117, China

**Keywords:** tea plant (*Camellia sinensis*), heat stress, trehalose, genome-wide identification, expression analysis

## Abstract

Heat stress exacerbated by global warming severely impairs the growth and tea quality of the tea plant (*Camellia sinensis*). Trehalose is pivotal for regulating plant growth and enhancing stress resistance. However, the molecular characteristics, expression patterns, and regulatory mechanisms of trehalose metabolism genes in tea plants under heat stress remain unclear. Therefore, this study conducted a comprehensive investigation of trehalose metabolism genes in the Tieguanyin tea plant genome. A total of 30 trehalose metabolism genes were identified, including 17 *trehalose-6-phosphate synthase* (*CsTPS*), 9 *trehalose-6-phosphate phosphatase* (*CsTPP*), and 4 *trehalase* (*CsTRE*) genes. These genes were characterized in terms of their chromosomal locations and gene structures; the encoded proteins were characterized in terms of their phylogenetic relationships, conserved motifs, functional domains, physicochemical properties, and subcellular distributions. The results showed that these genes exhibit family-specific structural and functional features, laying a foundation for further functional studies. Collinearity analysis identified 20 homologous gene pairs between tea plants and *Arabidopsis thaliana*, significantly more than the 3 pairs with *Oryza sativa*, suggesting a closer evolutionary relationship with *A. thaliana*. Additionally, five intraspecific duplicated gene pairs were identified, all with Ka/Ks values < 1, indicating they have undergone strong purifying selection during evolution, leading to functional stability. *Cis*-acting element analysis revealed abundant stress-responsive, light-responsive, and phytohormone-responsive elements in the promoter regions of these trehalose metabolism genes, indicating their potential involvement in tea plant stress resistance regulation. Differential expression analyses under heat stress with exogenous trehalose treatment (CK: control, T: water-sprayed heat stress, TT: 5.0 mM trehalose-sprayed heat stress) identified six differentially expressed genes (DEGs). We further analyzed the expression patterns of these DEGs. Specifically, *CsTPS1*, *CsTPS5*, and *CsTPS12* were increasingly upregulated in CK, T, and TT, respectively, while *CsTPP1* and *CsTPP2* were upregulated in TT relative to T. Additionally, *CsTRE1*, *CsTRE2*, and *CsTRE4* showed downregulation in TT compared to T, though they were not classified as DEGs. These findings indicate that exogenous trehalose application modulates trehalose metabolism by promoting *CsTPS* and *CsTPP* expression while inhibiting *CsTRE* expression, thereby increasing endogenous trehalose content in tea plants under heat stress. Yeast heat stress tolerance assays confirmed that *CsTPS1*, *CsTPS5*, *CsTPS12*, and *CsTPP1* enhanced yeast survival at 38 °C, verifying their function in improving organismal heat stress tolerance. In conclusion, these results clarify the roles of trehalose metabolism genes in tea plants’ heat stress response, demonstrating that exogenous trehalose modulates their expression to increase endogenous trehalose levels. This study provides a theoretical foundation for exploring trehalose-mediated heat stress resistance mechanisms and improving tea plant stress tolerance via genetic engineering.

## 1. Introduction

Heat stress, as one of the most severe abiotic stresses, poses a significant threat to plant growth and development. When exposed to high temperatures exceeding their optimal growth range, plants undergo a series of physiological, biochemical, and molecular changes that disrupt normal cellular functions [1,2]. Physiologically, heat stress can impair photosynthetic machinery, leading to reduced carbon assimilation and energy production [3]. It also induces excessive accumulation of reactive oxygen species (ROS), causing oxidative damage to lipids, proteins, and nucleic acids, which further compromises cell integrity and membrane stability [4]. At the molecular level, heat stress triggers the misfolding and denaturation of proteins, thereby activating stress-responsive signaling pathways. Various plants respond by upregulating the expression of heat shock proteins (HSPs), which act as molecular chaperones to assist in protein refolding and prevent aggregation [5,6,7]. However, prolonged or extreme heat stress can overwhelm these protective mechanisms, resulting in growth retardation, developmental abnormalities, and even plant death [8,9]. Moreover, heat stress disrupts a range of metabolic processes, such as carbohydrate metabolism [3], nitrogen metabolism [10] and redox metabolism [11], and these multifaceted impacts not only reduce crop production but also alter the quality of agricultural products [12]. Given the escalating frequency and intensity of heat waves due to global climate change, exploring eco-friendly protective agents that mediate plant responses to heat stress has become crucial for sustainable agricultural practices.

Trehalose, a non-reducing disaccharide, plays a pivotal role in plant responses to abiotic stresses such as heat and drought [13]. As a versatile biomolecule, it exhibits unique protective properties that enable plants to mitigate the detrimental effects of high-temperature exposure, including stabilizing cellular structures, maintaining osmotic equilibrium, activating key antioxidant enzymes, and effectively reducing oxidative damage under heat stress by scavenging ROS, among others [13,14,15]. For example, exogenous trehalose application confers high-temperature stress tolerance to herbaceous peony (*Paeonia lactiflora Pall.*) by enhancing antioxidant systems, activating photosynthesis, and protecting cell structure [16]. Similarly, in winter wheat (*Triticum aestivum*), it protects thylakoid membranes and photosynthetic capacity from heat-induced damage while reducing electrolyte leakage, malondialdehyde content, ROS levels, and lipoxygenase activity [17]. Additionally, trehalose functions as a signaling molecule that regulates the expression of antioxidant-related and stress-associated genes under heat stress conditions. Studies have demonstrated that it increases the expression of *SOD*, *CAT*, *POD* and *APX* genes in wheat [18] and genes encoding *vacuolar ATP synthase catalytic subunit A* in *A. thaliana* under high temperatures [10]. Furthermore, trehalose interacts with diverse pathways involving other signaling molecules (e.g., sugars, amino acids, phytohormones, polyamines) to regulate carbohydrate metabolism and secondary metabolism, thereby enhancing stress adaptation [13,19,20]. It has been reported that trehalose application enhanced chickpea (*Cicer arietinum* L.) seedling length and survival rate under heat stress, suggesting that trehalose acts downstream of abscisic acid (ABA) and partially modulates ABA-mediated protective responses [21]. Collectively, these findings highlight that trehalose enhances plant heat stress tolerance through a multi-faceted mechanism, and reveal its potential as a key regulator and practical tool for improving plant resilience under high-temperature conditions.

**Figure 1:**
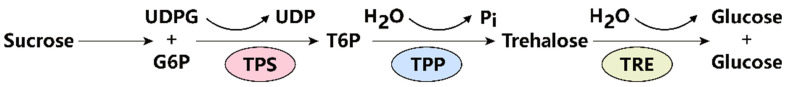
Trehalose metabolism in plants. Sucrose acts as the precursor, supplying uridine diphosphate glucose (UDPG) and glucose-6-phosphate (G6P) as substrates for trehalose-6-phosphate synthase (TPS). This enzyme catalyzes the formation of trehalose-6-phosphate (T6P), which is subsequently dephosphorylated into trehalose by trehalose-6-phosphate phosphatase (TPP). Finally, trehalose is hydrolyzed by trehalase (TRE) into two glucose molecules.

In plants, the biosynthesis and degradation of trehalose occur through a three-step reaction pathway (Figure 1). First, trehalose-6-phosphate synthase (TPS) catalyzes the formation of trehalose-6-phosphate (T6P) from UDP-glucose (UDPG) and glucose-6-phosphate (G6P). Subsequently, T6P is dephosphorylated to trehalose by trehalose-6-phosphate phosphatase (TPP). Finally, trehalase (TRE) hydrolyzes trehalose into two glucose (Glu) molecules [22,23]. These key enzymes, encoded by *TPS*, *TPP* and *TRE* genes, are critical for regulating trehalose levels, and their functions extend to modulating plant heat stress responses. For instance, transgenic *A. thaliana* expressing chimeric yeast *TPS* and *TPP* showed enhanced heat tolerance [24]. Transgenic alfalfa (*Medicago sativa* L.) expressing yeast *TPS1* and *TPP2* exhibited greater resilience to heat stress [25]. Similarly, transgenic tomatoes (*Solanum lycopersicum*) harboring *E. coli TPSP* (*trehalose-6-phosphate synthase/phosphatase*) accumulated more trehalose in seeds, leading to higher post-heat stress germination rates and faster induction of heat stress-responsive genes [26]. Conversely, CRISPR/Cas9-mediated knockout of *NtTRE* in tobacco (*Nicotiana tabacum*) increased heat stress sensitivity compared to wild-type plants [27]. Therefore, identifying trehalose metabolism genes and decoding their regulatory mechanisms are crucial for breeding heat-tolerant varieties.

Tea plant (*Camellia sinensis*) is important economic crops, and their growth and quality are highly susceptible to environmental stresses. Studies have shown that trehalose contributes to a series of physiological, biochemical, and molecular mechanisms that enable tea plants to mitigate the detrimental impacts of heat stress [28,29]. However, systematic identification and analysis of trehalose metabolism genes in tea plants remain lacking, and the effects of exogenous trehalose on the expression of these genes in tea plants under heat stress have not been reported. With the release of the Tieguanyin tea plant’s genome data, comprehensive genetic information has become available for the identification of trehalose metabolism genes. In this study, we identified the members of the *CsTPS*, *CsTPP* and *CsTRE* gene families in the Tieguanyin tea plant genome [30], and analyzed their genes at the levels of chromosomal locations, gene structure, collinearity, and cis-acting elements, as well as their encoded proteins at the levels of physicochemical properties, subcellular distributions, phylogenetic relationships, conserved motifs, and functional domains. We further investigated the expression patterns of *CsTPS*, *CsTPP* and *CsTRE* genes under heat stress and exogenous trehalose treatment, and yeast heat stress tolerance assays were conducted to examine the responses of *CsTPS1*, *CsTPS5*, *CsTPS12* and *CsTPP1* to heat stress. Our analysis aims to construct a regulatory network of trehalose metabolism genes in tea plants under heat stress, thereby providing crucial references for mechanistic research and practical applications in enhancing tea plant stress resistance.

## 2. Results

### 2.1. Identification and Physicochemical Property Analysis of Trehalose Metabolism Genes in Tea Plant

**Figure 2:**
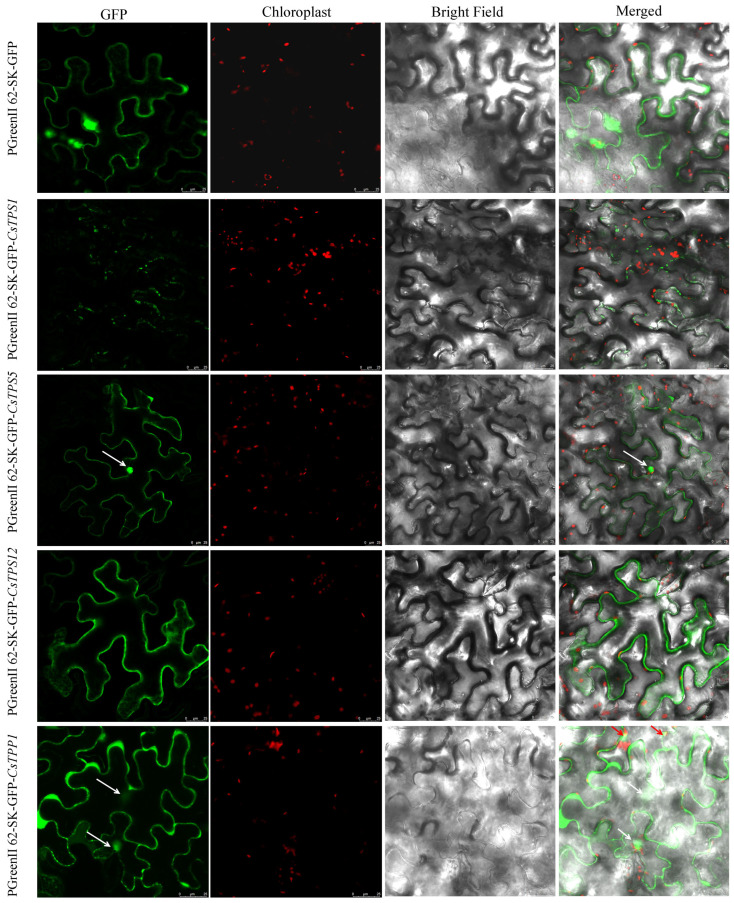
Subcellular localization of CsTPS1, CsTPS5, CsTPS12, and CsTPP1 proteins. Free GFP and GFP-fused recombinant proteins of CsTPS1, CsTPS5, CsTPS12, and CsTPP1 were transiently expressed in tobacco leaves and observed via fluorescence confocal microscopy. Red autofluorescence marks chloroplasts. White arrows indicate the nucleus, and red arrows point to chloroplast organelles.

In this study, a total of 30 trehalose metabolism genes were identified from the tea plant genome (*C. sinensis* cv. Tieguanyin), including 17 *CsTPSs*, nine *CsTPPs* and four *CsTREs*, which were then named based on their chromosomal locations (Table 1). To gain insight into their basic molecular characteristics, further analyses were performed on these identified genes and their encoded proteins. The encoded proteins’ physicochemical properties, such as the number of amino acids, isoelectric point (pI), molecular weight, instability index, aliphatic index, and grand average of hydropathicity (GRAVY), were analyzed. The sequences of trehalose metabolism proteins exhibited a wide range in length, from 109 amino acids (CsTPS10 and CsTPS16) to 1923 amino acids (CsTPP9), corresponding to molecular weights ranging from 12,647 Da to 220,197 Da. The pI values of the proteins also showed considerable diversity, with the lowest value of 4.72 detected in CsTPP7 and the highest of 9.84 in CsTPS8. Notably, CsTPS10, CsTPS16, and CsTPP6 had positive GRAVY values, suggesting a certain degree of hydrophobicity, while the remaining proteins had negative GRAVY values, indicating hydrophilic characteristics. The subcellular localization predictions indicated that trehalose metabolism proteins were distributed in various subcellular organelles, including the nucleus, chloroplast, peroxisome, mitochondrion, extracellular, and cytoplasm. In addition, CsTPS1, CsTPS5, CsTPS12, and CsTPP1 were selected for subcellular localization assays, with results presented in Figure 2. The results showed that CsTPS1 and CsTPS12 are cytoplasm-localized proteins, while CsTPS5 was demonstrated to localize in both the cytoplasm and nucleus, and CsTPP1 was found to be present in the cytoplasm, chloroplast, and nucleus. Notably, these experimental results do not always agree with the subcellular localization predictions. The observed discrepancies between the experimental results and the subcellular localization predictions may be attributed to the mismatch between the algorithmic limitations of prediction tools and the complexity of biological systems. Such single, dual, or multi-compartment localization patterns indicate that these proteins may perform distinct yet coordinated functions across subcellular compartments, participating in both stress responses and plant growth and development processes.

### 2.2. The Phylogenetic Analysis of Trehalose Metabolism Proteins

**Figure 3:**
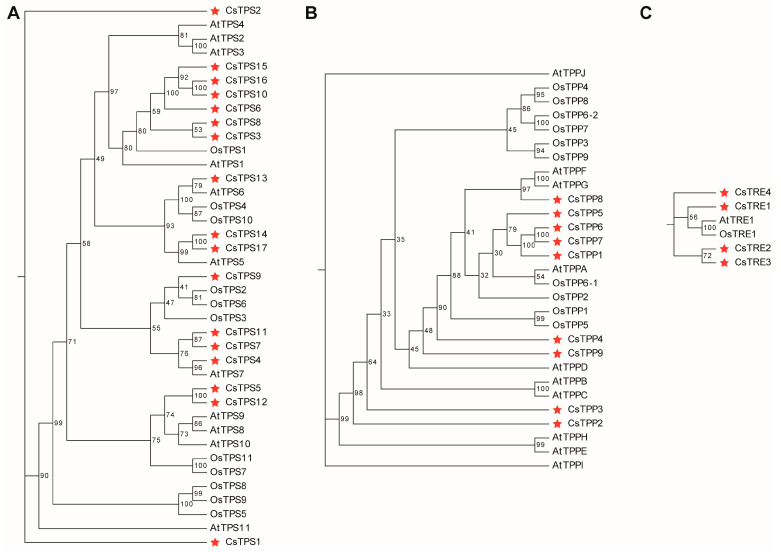
Phylogenetic analysis of TPS, TPP and TRE proteins from tea plant, *A. thaliana* and *O. sativa*. (**A**) Phylogenetic tree of TPS proteins. (**B**) Phylogenetic tree of TPP proteins. (**C**) Phylogenetic tree of TRE proteins. The phylogenetic tree was constructed using the One Step Build a Maximum Likelihood (ML) Tree method with 5000 bootstrap replicates in TBtools-II software (v2.363). The red star respsents the proteins in tea plant.

To gain insight into the evolutionary relationships of TPS, TPP, and TRE proteins in plants, phylogenetic trees were constructed using trehalose metabolism proteins from *A. thaliana*, *O. sativa* and *C. sinensis* (Figure 3). The results showed that the TPS category was the largest in the phylogenetic tree, containing a total of 39 members from the three plant species. In contrast, the TPP category included 29 members, and the TRE category was the smallest, with only six members. In the TPS category, CsTPS proteins formed multiple clades with TPS proteins from AtTPS and OsTPS. Most CsTPS members cluster among themselves. For instance, CsTPS15/16/10/6, CsTPS14/17, CsTPS11/7/4, and CsTPS5/12 clustered together with high bootstrap support (>75). However, CsTPS1 and CsTPS2 showed a relatively large divergence from other CsTPS members. Notably, CsTPS14/17 clustered with AtTPS5, CsTPS4 clustered with AtTPS7, and CsTPS9 clustered with OsTPS2/6, suggesting that these CsTPS genes are homologous with those these from *A. thaliana*, and *O. sativa* (Figure 3A). In the TPP protein category, CsTPP members (e.g., CsTPP5/6/7/1) formed clades with high bootstrap support values (>75). CsTPP8 clustered with AtTPPF/G, suggesting a close evolutionary relationship between the CsTPP8 and its homologs in *A. thaliana* (Figure 3B). In the TRE protein category, three clades were formed among these genes. CsTRE4 clustered independently, while CsTRE1, AtTRE1, and OsTRE1 clustered together. Additionally, CsTRE1 and CsTRE2 formed a separate cluster (Figure 3C). Taken together, the phylogenetic analysis indicated that tea plant CsTPS, CsTPP, and CsTRE proteins shared certain evolutionary relationships with their homologs in *A. thaliana* and *O. sativa*, alongside specific clustering patterns among individual members.

### 2.3. Phylogenetic Relationships, Conserved Motifs, and Functional Domains Analyses of CsTPS, CsTPP, and CsTRE Proteins, and Gene Structure Analysis of the CsTPS, CsTPP, and CsTRE Gene Families in Tea Plants

**Figure 4:**
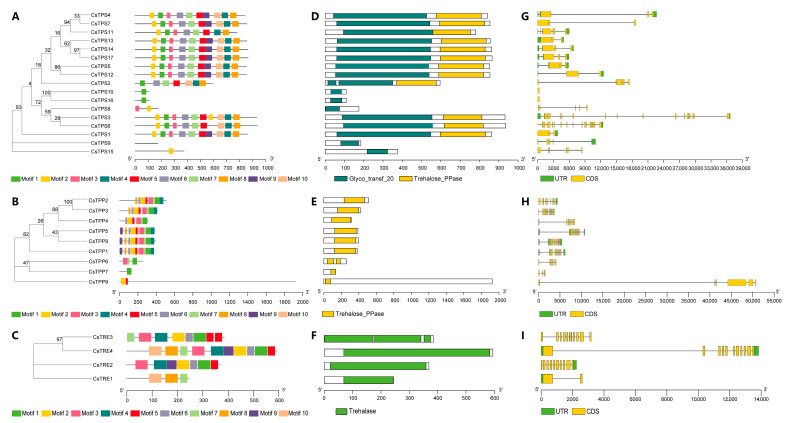
Phylogenetic relationships, conserved motifs, and functional domains analyses of CsTPS, CsTPP, and CsTRE proteins, and gene structure analysis of the *CsTPS*, *CsTPP*, and *CsTRE* gene families in tea plants. (**A**–**C**) Conserved motifs of CsTPS, CsTPP, and CsTRE proteins. (**D**–**F**) Functional domain of CsTPS, CsTPP, and CsTRE proteins. (**G**–**I**) Gene structure analysis of the *CsTPS*, *CsTPP*, and *CsTRE* gene families. The phylogenetic tree was constructed using the Neighbor-Joining method with 1000 bootstrap replicates in MEGA12 software (v12.0.9). The bootstrap consensus tree inferred from 1000 replicates was used to assess node support.

To analyze the sequential characteristics of trehalose metabolism proteins in tea plants, we identified their conserved domains and motif sequences. Motif analysis was conducted using MEME, and a total of 10 different motifs were identified in CsTPS, CsTPP, and CsTRE proteins, respectively. Among CsTPS proteins, eight members (CsTPS1, CsTPS4, CsTPS5, CsTPS7, CsTPS12, CsTPS13, CsTPS14, and CsTPS17) contained all 10 motifs. CsTPS10 and CsTPS16 possessed only motif 7, thus clustering together as a small branch, while CsTPS9 contained no motifs and CsTPS15 had only motif 2; consequently, these two formed separate branches distinct from other members (Figure 4A and Figure S1). For CsTPP proteins, more motif types were identified in CsTPP1, CsTPP2, CsTPP3, CsTPP4, CsTPP5, and CsTPP8, which clustered together in a large branch. In contrast, CsTPP6, CsTPS7, and CsTPP9 contained three, one, and two motif types, respectively, and, therefore, grouped separately (Figure 4B and Figure S1). Regarding CsTRE proteins, motif analysis showed that CsTRE4 contained all 10 motifs, while CsTRE2 and CsTRE3 each contained 7 motif types, and CsTRE1 had only 3 motif types (Figure 4C and Figure S1).

The conserved domain analysis revealed that all CsTPS proteins contained the characteristic Glyco_transf_20 domain, while most CsTPS proteins (except CsTPS8, CsTPS9, CsTPS10, CsTPS15, and CsTPS16) contained Trehalose_PPase domains (Figure 4D). Additionally, domain analysis of CsTPP proteins showed that all their sequences contained Trehalose_PPase domains, with CsTPS6 harboring two such domains (Figure 4E). For CsTRE proteins, domain analysis indicated that all members of the CsTRE family possessed a trehalase domain, while CsTRE3 contained three trehalase domains (Figure 4F).

Gene structure analysis revealed that members of the *CsTPS* gene family contained 1 to 18 exons. Among them, *CsTPS3* and *CsTPS6* had 18 exons along with a 5’UTR and a 3’UTR, forming a complete gene structure, while *CsTPS10* and *CsTPS16* had only one exon and lacked both a 5’UTR and a 3’UTR (Figure 4G). For the *CsTPP* gene family, the number of exons ranged from 4 to 14. *CsTPP5* and *CsTPP8* contained 14 exons, the highest number among *CsTPP* genes. In contrast, *CsTPP7* had the fewest exons (4) and lacked both a 5’UTR and a 3’UTR (Figure 4H). In the *CsTRE* gene family, *CsTRE1*, *CsTRE2*, *CsTRE3*, and *CsTRE4* contained 2, 10, 11, and 11 exons, respectively, with only *CsTRE4* exhibiting a complete gene structure (Figure 4I). These results suggested that there were differences in the length and composition of the 5’UTR, exons, and 3’UTR among different *CsTPS*, *CsTPP*, and *CsTRE* genes.

### 2.4. Collinearity Analysis of CsTPS, CsTPP, and CsTRE Genes

To better understand the potential evolutionary and functional relationships of trehalose metabolism genes across species, interspecific collinearity maps of tea plants with *Arabidopsis* and tea plants with rice were constructed (Figure S2). The results revealed 20 pairs of homologous genes between tea plants and *Arabidopsis*, a number significantly higher than the 3 pairs identified between tea plants and rice. These findings indicated that tea plants were more closely related to *Arabidopsis* in evolution than to rice, thus retaining more collinear homologous trehalose metabolism genes.

**Figure 5:**
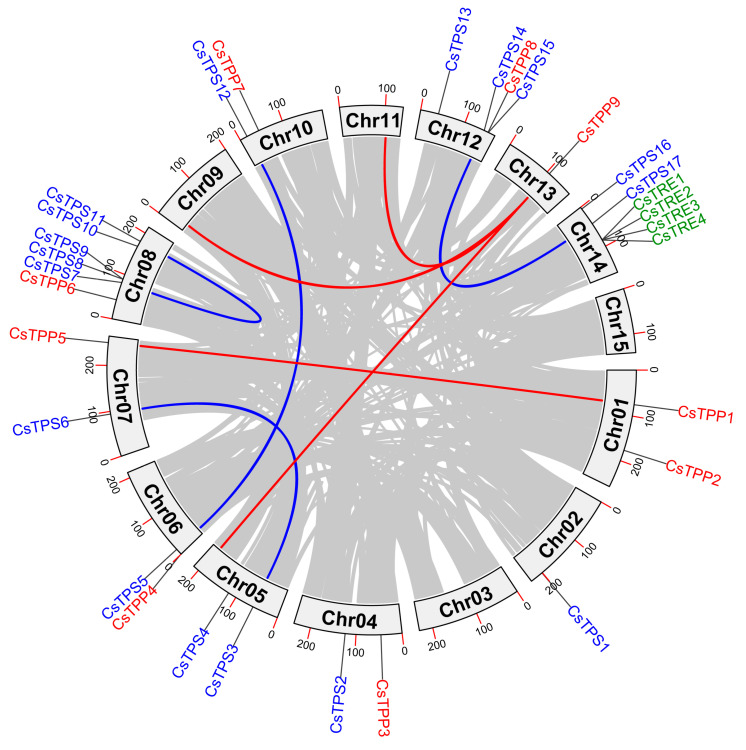
Intraspecific collinearity analysis of trehalose metabolism genes in *C. sinensis*. Gray lines denote collinear blocks in the tea plant genome; blue lines represent duplication events of *CsTPS* genes, and red lines represent duplication events of *CsTPP* genes. Red, blue, and green fonts denote the *CsTPP*, *CsTPS*, and *CsTRE* gene families, respectively.

To analyze the evolution of the *CsTPS*, *CsTPP* and *CsTRE* genes family in tea plant, gene duplication events and intraspecific collinearity were analyzed (Figure 5 and Supplementary Table S1). The analysis identified four collinear gene pairs within the *CsTPS* family, specifically *CsTPS3*-*CsTPS6*, *CsTPS5*-*CsTPS12*, *CsTPS7*-*CsTPS11*, and *CsTPS14*-*CsTPS17*. In contrast, only one duplicated gene pair (*CsTPP1*-*CsTPP5*) was detected in the *CsTPP* family. These observations supported that these identified genes may have originated from segmental duplication or whole-genome duplication (WGD) events. Notably, no collinear gene pairs were found in the *CsTRE* family; however, *CsTRE1* and *CsTRE2* are derived from proximal duplication, while *CsTRE4* arises from tandem duplication. The Ka/Ks ratios of these duplicated homologous pairs in the trehalose metabolism gene family were also calculated (Supplementary Table S1), all ranging from 0.160 to 0.306 and less than 1. This suggested that these gene pairs were subject to strong purifying selection during evolution, leading to their conserved and functionally stable characteristics.

### 2.5. Cis-Acting Element Analysis of CsTPS, CsTPP, and CsTRE Genes

**Figure 6:**
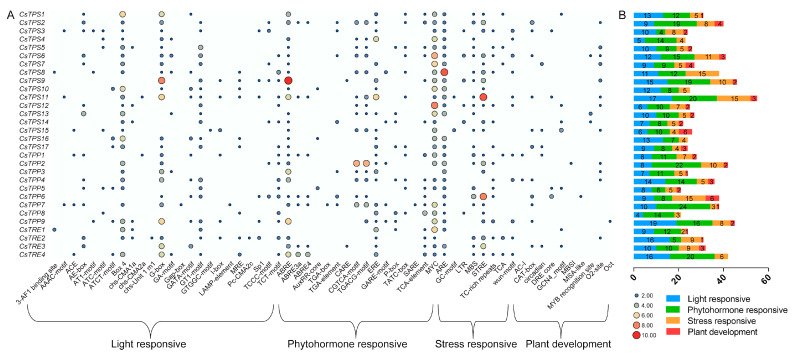
Analysis of the *cis*-acting elements in the promoters of *CsTPS*, *CsTPP* and *CsTRE* genes. (**A**) The *cis*-acting elements were classified into four categories: light responsiveness elements, phytohormone responsiveness elements, stress responsiveness elements, and plant development-related elements. (**B**) The histogram shows the number of *cis*-acting elements in each of the four categories, with different colors representing the respective categories.

To investigate the potential biological functions and transcriptional regulation of *CsTPS*, *CsTPP* and *CsTRE* genes in tea plants, the 2000 bp upstream genomic sequences of their start codons were analyzed using the PlantCARE online program (http://bioinformatics.psb.ugent.be/webtools/plantcare/html/, (accessed on 18 August 2025)). The identified *cis*-acting elements were categorized into four types: light responsiveness, phytohormone responsiveness, stress responsiveness, and plant development-related elements (Figure 6).

All *CsTPS*, *CsTPP* and *CsTRE* genes contained light-responsive elements in their promoter regions, indicating that their expression levels might be commonly regulated by light signaling. With respect to phytohormone-responsive elements, those corresponding to auxin (AuxRR-core, TGA-box, TGA-element), jasmonate (CGTCA-motif, TGACG-motif, ERE), gibberellin (GARE-motif, P-box, TATC-box), and salicylic acid (SARE, TCA-element) were predominantly present, though a few exceptions existed. For instance, *CsTPS3* lacked gibberellin- and salicylic acid-responsive elements, and *CsTPP5* lacked jasmonate-responsive elements. Stress-responsive elements (including ARE, GC-motif, LTR, MBS, STRE, TC-rich repeat, TCA, and wun-motif) were widely distributed, with all genes containing at least one such element. These suggested that the transcript abundance of these genes may be regulated by diverse biotic and abiotic stresses, with differences in element types potentially associated with their distinct functions under stress conditions. Among plant development-responsive elements, the main types were those involved in tissue-specific expression (e.g., O2_sites and GCN4 motifs) and circadian rhythm regulation (e.g., circadian), indicating that *CsTPS*, *CsTPP* and *CsTRE* genes play important roles in tea plant growth and development.

Overall, stress-responsive, light-responsive, and phytohormone-responsive elements were more prevalent than plant development-related elements in the promoters of these genes, highlighting their potential roles in responding to environmental cues and hormonal signals.

### 2.6. Expression Patterns of CsTPS, CsTPP, and CsTRE Genes Under Heat Stress with Exogenous Trehalose Treatment

**Figure 7:**
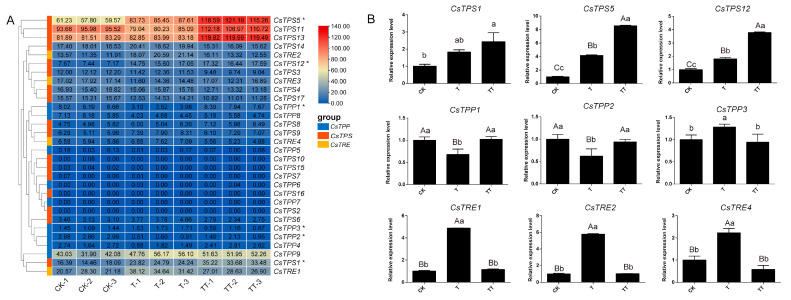
Expression levels of *CsTPS*, *CsTPP*, and *CsTRE* genes in CK (control group), T (sprayed with water and subjected to 24 h heat stress), and TT (sprayed with 5.0 mM trehalose and subjected to 24 h heat stress) samples. (**A**) Hierarchical clustering of expression levels of *CsTPS*, *CsTPP* and *CsTRE* genes in CK, T, and TT. Asterisks (*) indicate differentially expressed genes among the three samples (|log_2_FC| > 1 and *p*-value < 0.05). (**B**) Relative expression levels of *CsTPS1*, *CsTPS5*, *CsTPS12*, *CsTPP1*, *CsTPP2*, *CsTPP3*, *CsTRE1*, *CsTRE2*, and *CsTRE4* genes in CK, T, and TT samples. Lowercase letters indicate significant differences (*p* < 0.05), while uppercase letters indicate highly significant differences (*p* < 0.01).

The expression profiles of *CsTPS*, *CsTPP*, and *CsTRE* genes in CK, T, and TT samples were analyzed (Figure 7). These genes were classified according to their relative expression levels: seldom expressed (FPKM < 1), low (1–10 FPKM), medium (10–20 FPKM), high (20–50 FPKM), and extremely high (≥50 FPKM). Among these 30 genes, eight were seldom expressed, including *CsTPS2*, *CsTPS7*, *CsTPS10*, *CsTPS15*, *CsTPS16*, *CsTPP5*, *CsTPP6*, and *CsTPP7*. In contrast, *CsTPS5*, *CsTPS11*, and *CsTPS13* exhibited extremely high expression in the CK, T, and TT samples. It is noteworthy that among these trehalose metabolism genes, six (*CsTPS1*, *CsTPS5*, *CsTPS12*, *CsTPP1*, *CsTPP2*, and *CsTPP3*) were differentially expressed. Their expression patterns showed that *CsTPS1*, *CsTPS5*, and *CsTPS12* displayed increased expression in CK, T, and TT samples and were differentially expressed in the TT-vs-CK comparison group. Within the *CsTPP* gene family, *CsTPP1* and *CsTPP2* exhibited significantly increased expression in TT samples compared with T samples, while *CsTPP3* showed decreased expression in TT samples (Figure 7A).

To validate these transcriptome-based findings, the six DEGs (*CsTPS1*, *CsTPS5*, *CsTPS12*, *CsTPP1*, *CsTPP2*, *CsTPP3*) along with *CsTRE1*, *CsTRE2*, and *CsTRE4* (non-DEGs) were selected for RT-qPCR analysis. The results confirmed the expression trends: *CsTPS1*, *CsTPS5*, and *CsTPS12* were upregulated in CK, T, and TT samples; *CsTPP1* and *CsTPP2* were significantly increased in TT samples compared to T samples; and, conversely, *CsTRE1*, *CsTRE2*, and *CsTRE4* were significantly decreased in TT samples compared to T samples (Figure 7B). These consistent expression patterns suggest that exogenous trehalose upregulates the *CsTPS* and *CsTPP* genes families while downregulating *CsTRE* genes, thereby promoting endogenous trehalose accumulation.

### 2.7. Analysis of CsTPS1, CsTPS5, CsTPS12, and CsTPP1 Response to Heat Stress

**Figure 8:**
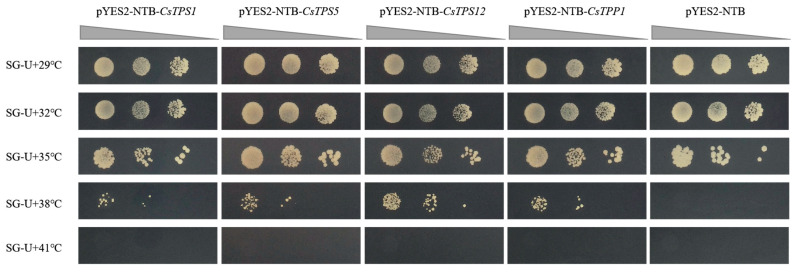
Growth activity of yeast expressing *CsTPS1*, *CsTPS5*, *CsTPS12*, or *CsTPP1* under different temperature conditions. Yeast strain BY4741 was transformed with pYES2-NTB-*CsTPS1*, pYES2-NTB-*CsTPS5*, pYES2-NTB-*CsTPS12*, pYES2-NTB-*CsTPP1*, or the empty pYES2-NTB vector (control), respectively. Transformants were grown on SG-Ura medium at 29 °C, 32 °C, 35 °C, 38 °C, and 41 °C. The dilution concentrations of the yeast liquid are as follows: 0.5, 0.05, and 0.005 (from left to right).

To characterize the regulatory roles of *CsTPS1*, *CsTPS5*, *CsTPS12*, and *CsTPP1* in response to heat stress, we evaluated the growth of *Saccharomyces cerevisiae* cells harboring these genes under different temperature conditions. The results showed that yeast cells expressing *CsTPS1*, *CsTPS5*, *CsTPS12*, or *CsTPP1*, as well as the control strain (PYES2-NTB), survived well at 29 °C, 32 °C, and 35 °C. However, at 38 °C (heat stress treatment), yeast cells expressing *CsTPS1*, *CsTPS5*, *CsTPS12*, or *CsTPP1* exhibited a higher survival rate than the control (PYES2-NTB) when grown on SC-Ura medium (Figure 8). These findings suggest that *CsTPS1*, *CsTPS5*, *CsTPS12*, and *CsTPP1* are involved in the heat stress response.

## 3. Discussion

### 3.1. Structural Characteristics and Molecular Evolution of Trehalose Metabolism Proteins in Tea Plants

As an osmolyte, energy reservoir, and signaling molecule, trehalose contributes to the coordination of plants’ adaptive responses when facing temperature stress [15]. TPS and TPP are key enzymes in the trehalose synthesis pathway, while TRE is the enzyme that catalyzes the biological decomposition of trehalose. Therefore, studying the characteristics and functions of TPS, TPP, and TRE will contribute to understanding the mechanisms of trehalose metabolism in plants during heat stress. Trehalose metabolism (TPS, TPP, and TRE) is ubiquitous in plants, and the number of these genes varies across different plant species. For example, *Arabidopsis thaliana* possesses 11 *AtTPS*, 10 *AtTPP*, and 1 *AtTRE* genes [31]; 11 *SlTPS*, 8 *SlTPP*, and 1 *SlTRE* genes have been identified in tomato [32]; and 11 *OsTPS*, 10 *OsTPP*, and 1 *OsTRE* genes have been identified in rice [33,34]. In this study, a total of 17 *CsTPS*, 9 *CsTPP*, and 4 *CsTRE* genes were identified in the genome of tea plant. The significant variation in the number of these gene family members across plant species suggests that gene duplication and loss events likely occurred in these families during evolution. Notably, tea plants possess more *CsTPS* and *CsTRE* genes than *A. thaliana*, tomato, and rice. This difference may be attributed to the larger genome size of tea plants (3.15 Gb) compared to that of *A. thaliana* (125 Mb), tomato (950 Mb), and rice (430 Mb), as well as potential expansion events of *CsTPS* and *CsTRE* genes in tea plants.

To investigate the molecular characteristics of trehalose metabolism proteins in tea plants, the diverse physicochemical properties (e.g., amino acid length, isoelectric point, molecular weight), subcellular localizations, and chromosomal distributions were analyzed (Table 1 and Figure 2). These results not only reflect the distinct characteristics of the three gene families (*CsTPS*, *CsTPP*, and *CsTRE*) but also provide insights into their functional specialization. Specifically, the differential subcellular localizations correspond to their potential functional roles. Proteins localized in chloroplasts may directly participate in photosynthesis-related trehalose metabolism [35]; those in the cytoplasm likely play key roles in processes such as protein folding and maintenance of cellular homeostasis through trehalose-mediated protection [36], while nuclear-localized proteins might interact with transcription factors or chromatin-associated proteins to regulate the expression of heat stress-responsive genes [10,18,37]. In this study, our subcellular localization assay revealed that trehalose metabolism-related proteins are not concentrated in a single specific compartment but rather widely distributed across multiple subcellular organelles. This broad distribution pattern supports the existence of a coordinated regulatory network, where these proteins act synergistically across different organelles to modulate trehalose metabolism and integrate cellular responses to heat stress.

Phylogenetic tree analysis indicated that TPS, TPP, and TRE proteins in tea plant shared evolutionary conservation with their homologs in *A. thaliana* and *O. sativa*, while obvious subfamily differentiation existed among tea plant’s own family members, reflecting a combination of species specificity and evolutionary relatedness (Figure 3). In addition, interspecific collinearity analysis revealed that more duplicated homologous trehalose metabolism gene pairs were detected in two dicots (*A. thaliana* and *C. sinensis*) than in monocot-dicot (*O. sativa* and *C. sinensis*), indicating these genes may have experienced expansion events after the divergence of monocots and dicots (Figure S2). Conserved domain analysis of tea plant trehalose metabolism proteins showed that all CsTPS proteins contain the Glyco_transf_20 domain, with most (except CsTPS8/9/10/15/16 members) additionally harboring Trehalose_PPase domains; all CsTPP proteins possess Trehalose_PPase domains; and every member of the CsTRE family carries a trehalase domain (Figure 4D–F). This observation aligns with findings in other plant species that TPS proteins typically contain two key domains (Glyco_transf_20 and Trehalose_PPase); TPP proteins consist solely of the Trehalose_PPase domain; and trehalase proteins harbor the trehalase domain [38,39,40]. Although both TPP and TPS proteins in plants contain Trehalose_PPase domains, the Trehalose_PPase domain in TPS proteins has lost its enzymatic function during evolution [33]. Collectively, these results confirmed that the typical domains of the trehalose metabolism gene family exhibit functional conservation across different plant species. Moreover, five duplicated gene pairs involved in trehalose metabolism have been identified in tea plants (Figure 5), all of which originated from WGD or segmental duplication events (Supplementary Table S2). Additionally, all Ka/Ks ratios of these duplicated gene pairs are below 1.0 (Supplementary Table S2). This not only confirms that the gene pairs originated from WGD or segmental duplication but also indicates they are subject to strong purifying selection, with this selection mechanism preserving the functional conservation of the duplicated pairs.

In summary, gene duplication events are widespread in plants and serve as a crucial driving force behind changes in genomic gene number, the emergence of new gene functions, and gene rearrangements. For tea plant trehalose metabolism genes specifically, multiple gene replication types are not only the main driver of their family expansion but also the prerequisite for promoting functional differentiation among these genes. Against this background, the differences in trehalose metabolism genes at the level of gene structure, as well as the differences in their encoded proteins at the levels of domain and motif composition, may further accelerate the functional diversification of the duplicated gene members. These factors synergistically drive a clear division of labor among trehalose metabolism genes; as a result, these genes exert distinctly different roles in various tea plant tissues and during environmental adaptation, which may further underpin the formation of a precise trehalose metabolism regulatory mechanism in tea plants.

### 3.2. Trehalose Metabolism Genes Play a Crucial Role in Tea Plants’ Response to Heat Stress

Increasing evidence indicates that trehalose metabolism genes play an important role in responding to various environmental stresses. For example, in rice, overexpression of the *OsTPP3* enhances the plant’s drought tolerance compared to wild-type plants [41]; similarly, under chilling stress, the *OsTPP1* is upregulated by the phosphorylated *OsbHLH002* (triggered by active *OsMAPK3*), which increases trehalose content and thereby improves chilling damage resistance in rice [42]. In *A. thaliana*, heterologous overexpression of watermelon (*Citrullus lanatus*) *ClTPS3* significantly boosts salt tolerance of the transgenic plants [43]. For *Pleurotus ostreatus* mycelia, under heat stress, overexpression of *TPS* and *TRE* leads to increased and decreased trehalose levels, respectively [44]. Additionally, transgenic tomato plants expressing the *E. coli TPSP* gene exhibit enhanced trehalose content in seeds, higher germination rates after heat stress, and faster expression of heat stress-responsive genes compared to wild-type tomatoes [26]. In our study, the identification of numerous stress response-related *cis*-acting elements in the promoter regions of trehalose metabolism genes implies that these genes are also extensively involved in stress responses. Specifically, LTR *cis*-acting elements (associated with low-temperature responsiveness) are predominantly distributed in *TPS* genes, and most trehalose metabolism genes contain MBS and TC-rich repeats cis-acting elements, which are involved in drought-inducibility and defense/stress responsiveness, respectively (Figure 6).

In addition, the expression patterns of genes involved in trehalose metabolism showed that *CsTPS1*, *CsTPS5*, and *CsTPS12* were increasingly upregulated across CK, T, and TT samples, respectively. Compared with the T sample, the TT sample exhibited increased expression of *CsTPP1* and *CsTPP2*, whereas *CsTRE1*, *CsTRE2*, and *CsTRE4* were downregulated (Figure 7). Since TPS and TPP are key enzymes in trehalose biosynthesis, while TRE catalyzes the hydrolysis of trehalose into two glucose molecules, the coordinated upregulation of *TPS* and *TPP* genes together with the downregulation of *TRE* genes in the TT sample suggests that exogenous trehalose treatment can alter the expression of trehalose metabolism genes, thereby contributing to increased endogenous trehalose levels under heat stress. This is consistent with previous findings showing that exogenous trehalose application significantly increases endogenous trehalose content in plants exposed to heat stress [28]. Consistent with these results, exogenous trehalose application significantly upregulated TPS enzyme activity in *Gracilaria lemaneiformis* [45]. Similarly, in rapeseed *(Brassica napus* L.) seedlings exposed to cold stress (4 and –4 °C), treatment with trehalose markedly increased the transcription levels of trehalose-biosynthetic genes, including *TPS4*, *TPS8*, and *TPS9* [46]. Likewise, priming wheat seeds with trehalose upregulated the expression of *TPP1* and *TPP2* under chilling stress (15 °C) [47].

Furthermore, to characterize the regulatory roles of *CsTPS1*, *CsTPS5*, *CsTPS12*, and *CsTPP1* in heat stress response, we found that yeast cells expressing these genes exhibited a higher survival rate than the control (Figure 8). This result indicates that *CsTPS1*, *CsTPS5*, *CsTPS12*, and *CsTPP1* play important roles in mediating heat stress tolerance.

## 4. Materials and Methods

### 4.1. Plant Materials and Heat Stress Treatment

*C*. *sinensis* cv. Tieguanyin was planted in the tea garden of Ningde Normal University, Ningde, China (26°39′40″ N, 119°35′6″ E). Two-year-old tea plants (*C. sinensis* cv. Tieguanyin) were first acclimated in an artificial climate incubator under a 12 h light (25 °C)/12 h darkness (19 °C) cycle with 75% relative humidity for 7 days. They were then subjected to heat stress under a 12 h light (38 °C)/12 h darkness (29 °C) cycle (75% humidity). After heat pretreatment, samples were collected as the control group (CK), while the remaining plants were either sprayed with water and subjected to 24 h heat stress (T) or sprayed with 5.0 mM trehalose and subjected to 24 h heat stress (TT) [28]. Three independent biological replicates were performed for each treatment (CK, T, and TT). For each replicate, one bud with the second and third leaves was collected from the top and lateral branches of an individual plant, immediately frozen in liquid nitrogen, and stored at −80 °C for subsequent analyses.

### 4.2. Identification and Characterization of Trehalose Metabolism Genes in Tea Plant

To identify trehalose metabolism genes in the Tieguanyin tea plant genome, sequences of *AtTPS*, *AtTPP* and *AtTRE* genes from *A. thaliana* were downloaded from the TAIR database (www.arabidopsis.org/index.jsp, (accessed on 10 March 2025)) as references. Potential *CsTPS*, *CsTPP* and *CsTRE* genes were retrieved by BLASTP search (with a *p*-value < 1 × 10^−5^) against the Tieguanyin tea plant genome using TBtools software [48].

The ProtParam tool (https://www.expasy.org/ (accessed on 12 May 2025)) was employed to analyze the isoelectric point (pI) and molecular weight (MW) of the proteins encoded by *CsTPS*, *CsTPP* and *CsTRE* genes. The WoLF PSORT server (https://wolfpsort.hgc.jp/ (accessed on 10 May 2025)) was used to predict the subcellular localization of the related proteins.

### 4.3. Subcellular Localization Assay

The open reading frames of *CsTPS1*, *CsTPS5*, *CsTPS12*, and *CsTPP1* were each cloned into the pGreenII 62-SK-GFP vector, with sequencing validation to confirm correct insertion; the GFP-fused vectors were then transformed into *Agrobacterium tumefaciens* GV3101 (WeiDi, Shanghai, China). Transformed Agrobacteria were plated on LB agar containing kanamycin (50 μg/mL) and rifampicin (25 μg/mL) and incubated at 28 °C for 2–3 days; single positive colonies were then picked and cultured in LB liquid medium (supplemented with the same antibiotics) at 28 °C and 220 rpm for 36–48 h. Agrobacterial cells were centrifuged (7000 rpm, 2 min), washed with MES, resuspended in MES to OD_600_ ≈ 1.4, mixed with acetosyringone (150 μM final), and incubated at 28 °C, 220 rpm for 1–1.5 h. The prepared bacterial suspension was infiltrated into the abaxial side of 4–5-week-old *Nicotiana benthamiana* leaves, which was provided by the Institute of Horticultural Biotechnology, Fujian Agriculture and Forestry University, Fuzhou, China. Infiltrated plants were cultured at 25 °C for 36–48 h; afterward, leaf sections were mounted on slides, and fluorescence signals were observed using a Leica TCS SP8 confocal microscope (Leica Microsystems GmbH, Wetzlar, Germany) with the following settings: GFP (excitation wavelength: 488 nm, emission wavelength: 495–523 nm) and chloroplast autofluorescence (excitation wavelength: 542 nm, emission wavelength: 650–670 nm).

### 4.4. Phylogenetic Analysis of Trehalose Metabolism Genes in Tea Plant

Phylogenetic analysis of TPS, TPP, and TRE proteins from tea plant, *A. thaliana*, and *O. sativa* was performed using the maximum likelihood (ML) method. The phylogenetic tree was constructed via the One Step Build a ML Tree method with 5000 bootstrap replicates in TBtools software. Additionally, phylogenetic relationships of CsTPS, CsTPP, and CsTRE proteins were constructed using the Neighbor-Joining method in MEGA12 with a bootstrap value of 1000 [49]. Detailed protein sequences from *A. thaliana* and *O. sativa* are listed in Supplementary Table S3, with those of *O. sativa* downloaded from the RAP-DB database (https://rapdb.dna.affrc.go.jp/, (accessed on 11 July 2025)).

### 4.5. Conserved Motifs, Functional Domains of Trehalose Metabolism Proteins, and Gene Structure Analysis of Trehalose Metabolism Genes in Tea Plants

The conserved motifs of the CsTPS, CsTPP and CsTRE protein sequences in tea plant were predicted through the MEME online program with maximum number of motifs = 10 and the other parameters using default settings. The conserved domains of their sequences were analyzed using the Simple Modular Architecture Research Tool (SMART, http://smart.embl-heidelberg.de/, (accessed on 11 August 2025)). Based on the genome annotation files of the Tieguanyin tea plant, the gene structures of *CsTPS*, *CsTPP* and *CsTRE* genes were visualized using the TBtools software.

### 4.6. Collinearity Analysis of Trehalose Metabolism Genes in Tea Plant

The TBtools software was used to conduct both inter-species collinearity analysis of trehalose metabolism genes among *C. sinensis*, *A. thaliana*, and *O. sativa*, and intraspecific synteny analysis in *C. sinensis*. The analytical process involved two main steps: first, retrieving genome sequences and annotation files of the aforementioned species; second, conducting chromosome structure and collinearity analyses based on the GFF files of these species’ genomes.

### 4.7. Cis-Acting Elements Analysis of Trehalose Metabolism Genes in Tea Plant

The promoter regions of trehalose metabolism genes, defined as the 2000 bp nucleotide sequences upstream of their translation start sites, were extracted using TBtools software. These sequences were then submitted to the PlantCARE online program (http://bioinformatics.psb.ugent.be/webtools/plantcare/html/, (accessed on 8 August 2025)) for prediction and identification of putative *cis*-acting elements [50]. The identified *cis*-acting elements were summarized and analyzed, and the results were visualized using TBtools software.

### 4.8. Expression Pattern Analysis of Trehalose Metabolism Genes in Tea Plant

Gene expression levels were quantified using the Fragments per Kilobase of transcript per Million mapped reads (FPKM) method [51]. The FPKM expression profiles of trehalose metabolism genes in CK, T, and TT samples were extracted from their respective transcriptome datasets, which were retrieved from the Sequence Read Archive (SRA) database (accession number: PRJNA1178683). After obtaining high-quality clean reads, HISAT2 [52] were used to align clean reads to the reference genome of the Tieguanyin tea plant genome. The estimateSizeFactors function of the DESeq (2012) R [53] package (v.24.0) was used to normalize the counts, and the nbinomTest function was applied to calculate the *p*-values and fold change values for differential comparison (T-vs-CK, TT -vs-CK, TT-vs-T). Finally, genes with |log_2_FC| > 1 and *p*-value < 0.05 were identified as differentially expressed genes (DEGs). The expression patterns of these trehalose metabolism genes were visually depicted by a heatmap generated using TBtools software.

### 4.9. Quantitative Real-Time PCR (RT-qPCR) Analysis

For RT-qPCR analysis, total RNA was extracted from the samples, and first-strand cDNA was synthesized using the Hifair^®^ 1st Strand cDNA Synthesis kit (Yeasen, Shanghai, China). Primers for RT-qPCR were designed using the NCBI Primer designing tool (https://www.ncbi.nlm.nih.gov/tools/primer-blast/, (accessed on 22 May 2025)) based on the cDNA sequences (Supplementary Table S4). Reactions were run on a Roche LightCycler 480 instrument in a 20 μL system: 10 μL Hieff^®^ qPCR SYBR^®^ Green Master Mix (Yeasen), 0.8 μL each primer, 1.0 μL 10-fold diluted cDNA, and 7.4 μL ddH_2_O. The relative expression levels of the genes were normalized to reference gene *GAPDH*, and calculated using the 2^−ΔΔCT^ algorithm. All analyses were performed with three independent biological replications, and data were presented as mean ± standard deviation (SD).

### 4.10. Heat Stress Tolerance Assays of CsTPS1, CsTPS5, CsTPS12 and CsTPP1 in Yeast

The full-length coding sequences of *CsTPS1*, *CsTPS5*, *CsTPS12* and *CsTPP1* were synthesized via chemical oligonucleotide synthesis and inserted into the pYES2-NTB vector using *NcoI*/*BamHI* restriction enzymes. These constructs (pYES2-NTB-*CsTPS1*, pYES2-NTB-*CsTPS5*, pYES2-NTB-*CsTPS12* and pYES2-NTB-*CsTPP1*) were then transformed into the *Saccharomyces cerevisiae* strain BY4741 (Miaoling, Wuhan, China), respectively. The pYES2-NTB plasmid was used as a negative control. For yeast transformation, a single BY4741 colony from YPDA plates was cultured in 4 mL YPDA liquid medium (1% Yeast extract, 2% Peptone, 2% Glucose and 0.02% Adenine) overnight (30 °C, 225 rpm) until OD600 > 1.5, then transferred to 50 mL YPDA to an initial OD600 = 0.2 and cultured for 4–5 h until OD600 = 0.6. Cells were harvested by centrifugation (4000 rpm, 5 min, room temperature), washed with sterile water and 0.1 M LiOAc sequentially, then resuspended in 500 μL 0.1 M LiAc and aliquoted (50 μL per transformation). For each transformation, 240 μL 50% PEG3350, 36 μL 1 M LiAc, 5 μL ssDNA (10 mg/mL), and 5 μL plasmid DNA were added, mixed thoroughly, incubated at 30 °C for 30 min, heat-shocked at 42 °C for 25 min, and recovered at 30 °C for 30 min. After centrifugation, cells were resuspended in 200 μL sterile water, spread on defective screening plates, and cultured at 30 °C for 4 days.

Eight colonies were randomly selected from transformed plates for PCR verification, and positive clones were used for phenotypic analysis. Correct positive clones of experimental groups (pYES2-NTB-*CsTPS1*, pYES2-NTB-*CsTPS5*, pYES2-NTB-*CsTPS12* and pYES2-NTB-*CsTPP1*) and negative control (pYES2-NTB) were resuspended in 2 mL ddH_2_O to adjust OD600 to 0.5, spotted on SG-Ura (Synthetic Galactose Minimal Medium without Uracil) plates, and incubated at 29 °C, 32 °C, 35 °C, 38 °C, and 41 °C. Plates were observed and photographed after 7 days.

## 5. Conclusions

This study identified and characterized 30 trehalose metabolism genes (17 *CsTPS*, nine *CsTPP*, four *CsTRE*) from the Tieguanyin tea plant genome, clarifying their molecular features, evolutionary relationships, and heat stress response roles. These genes exhibited diverse physicochemical properties and subcellular localizations. Phylogenetic analysis revealed that tea plant CsTPS, CsTPP, and CsTRE proteins shared certain evolutionary relationships with their homologs in *A. thaliana* and *O. sativa*, with distinct clustering patterns among individual members. Interspecific collinearity analysis showed more duplicated homologous trehalose metabolism gene pairs in dicots (*A. thaliana* and *C. sinensis*) than in monocot-dicot (*O. sativa* and *C. sinensis*), suggesting that these genes expanded after monocots and dicots diverged. Five intraspecific duplicated gene pairs derived from whole-genome/segmental duplication undergo strong purifying selection, indicating functional conservation and supporting family expansion-driven adaptive evolution. Promoters of these genes contain numerous stress-responsive *cis*-elements, pointing to their involvement in stress adaptation. Expression analysis confirmed that exogenous trehalose treatment upregulates *CsTPS* and *CsTPP* and downregulates *CsTRE*, thereby promoting endogenous trehalose accumulation. Yeast assays verified that *CsTPS1*, *CsTPS5*, *CsTPS12*, and *CsTPP1* enhance heat tolerance. Collectively, this study establishes a foundational understanding of tea plant trehalose metabolism genes and their heat stress regulatory network, providing references for trehalose-mediated stress resistance research and tea plant heat tolerance improvement.

## Figures and Tables

**Table 1:** Trehalose metabolism genes in tea plant.

Genome ID	Gene Name	Location on Chromosome			Amino Acid (aa)	Isoelectric Point	Molecular Weight	Instability Index	Aliphatic Index	Grand Average of Hydropathicity	Predicted Subcellular Location
		Chr	Start	End							
CsTGY02G0002941	*CsTPS1*	Chr02	200,749,218	200,753,015	862	5.84	97,862	47.91	84.19	−0.269	chloroplast
CsTGY04G0001703	*CsTPS2*	Chr04	123,640,109	123,657,586	596	7.03	66,845	39.54	87.75	−0.19	nucleus
CsTGY05G0000915	*CsTPS3*	Chr05	57,599,302	57,635,952	932	7.18	104,904	43.99	85.46	−0.357	peroxisome
CsTGY05G0001584	*CsTPS4*	Chr05	118,346,295	118,368,930	842	5.54	95,441	48	83.47	−0.292	nucleus
CsTGY06G0000336	*CsTPS5*	Chr06	14,036,327	14,042,199	854	6.06	96,663	47.8	90.13	−0.192	cytoplasm
CsTGY07G0000650	*CsTPS6*	Chr07	94,274,222	94,286,663	935	6.57	104,640	39.85	85.29	−0.343	mitochondrion
CsTGY08G0000966	*CsTPS7*	Chr08	81,319,196	81,337,863	855	6.32	96,540	45	87.33	−0.183	chloroplast
CsTGY08G0001044	*CsTPS8*	Chr08	88,871,360	88,880,830	175	9.84	19,913	43.84	98.17	−0.329	cytoplasm
CsTGY08G0001045	*CsTPS9*	Chr08	88,893,568	88,904,580	185	6.44	21,360	63.25	84.86	−0.465	chloroplast
CsTGY08G0001896	*CsTPS10*	Chr08	166,945,078	166,945,407	109	8.23	12,687	49.69	76.06	0.236	chloroplast
CsTGY08G0002084	*CsTPS11*	Chr08	179,049,653	179,055,642	781	5.67	87,823	45.4	82.84	−0.273	nucleus
CsTGY10G0000343	*CsTPS12*	Chr10	16,604,285	16,616,852	855	5.77	96,447	49.52	89.71	−0.149	cytoplasm
CsTGY12G0000461	*CsTPS13*	Chr12	39,489,896	39,494,837	856	5.65	97,350	41.47	91.51	−0.207	nucleus
CsTGY12G0001545	*CsTPS14*	Chr12	144,915,194	144,922,004	863	5.59	97,244	44.3	92.11	−0.164	chloroplast
CsTGY12G0001771	*CsTPS15*	Chr12	154,839,943	154,848,421	376	9.24	41,499	40.56	87.95	−0.031	chloroplast
CsTGY14G0000106	*CsTPS16*	Chr14	4,531,582	4,531,911	109	8.65	12,647	48.42	76.06	0.188	chloroplast
CsTGY14G0000925	*CsTPS17*	Chr14	43,647,873	43,653,809	865	5.7	97,429	50.75	91.82	−0.187	cytoplasm
CsTGY01G0001161	*CsTPP1*	Chr01	75,066,031	75,072,244	382	8.98	42,683	38.31	82.88	−0.388	nucleus
CsTGY01G0002203	*CsTPP2*	Chr01	176,281,234	176,285,628	510	9.35	57,780	38.93	80.27	−0.414	cytoplasm
CsTGY04G0000901	*CsTPP3*	Chr04	46,012,012	46,015,699	418	9.02	46,615	29.76	85.38	−0.376	nucleus
CsTGY06G0000007	*CsTPP4*	Chr06	381,529	389,971	314	6.22	36,052	36.95	75.45	−0.545	mitochondrion
CsTGY07G0002823	*CsTPP5*	Chr07	249,344,114	249,354,882	390	6.33	43,897	35.02	84.21	−0.339	nucleus
CsTGY08G0000603	*CsTPP6*	Chr08	42,227,240	42,231,370	257	5.57	29,498	30.59	117.04	0.14	nucleus
CsTGY10G0000716	*CsTPP7*	Chr10	44,849,190	44,850,744	133	4.72	15,070	24.06	106.09	−0.165	cytoplasm
CsTGY12G0001751	*CsTPP8*	Chr12	154,099,590	154,105,064	396	7.56	44,474	35.38	84.87	−0.354	nucleus
CsTGY13G0001345	*CsTPP9*	Chr13	106,148,166	106,198,927	1923	4.99	220,197	51.61	85.67	−0.705	cytoplasm
CsTGY14G0001620	*CsTRE1*	Chr14	84,599,742	84,602,355	244	6.91	27,556	27.41	94.59	−0.037	chloroplast
CsTGY14G0001623	*CsTRE2*	Chr14	84,614,556	84,616,810	369	5.44	41,729	39.05	77.75	−0.214	nucleus
CsTGY14G0001626	*CsTRE3*	Chr14	84,879,286	84,882,465	385	5.05	43,282	39.87	82.62	−0.192	extracellular
CsTGY14G0001627	*CsTRE4*	Chr14	85,103,603	85,117,441	594	5.49	66,878	36.26	84.39	−0.169	extracellular

## Data Availability

All data presented in this study are provided either in the manuscript or Supplementary Materials.

## Supplementary Materials

The following supporting information can be downloaded at: https://www.mdpi.com/article/10.3390/plants14213309/s1, Figure S1. Conserved motif identification of CsTPS, CsTPP, and CsTRE proteins. Figure S2. Interspecific collinearity of trehalose metabolism genes between *C. sinensis* and *A. thaliana*, and between *C. sinensis* and *O. sativa*. Blue lines represent all collinear relationships of trehalose metabolism genes between tea plants and the other two species, while gray lines indicate genome-wide collinear blocks between the respective species. Table S1. Gene duplication event analysis of trehalose metabolic genes. Table S2. Parameters of duplication events of the trehalose metabolic gene pairs. Table S3. Detail information of trehalose metabolism genes used for the construction of phylogenetic trees. Table S4. List of primers used in this study.

## References

[1] Djalovic I., Kundu S., Bahuguna R.N., Pareek A., Raza A., Singla-Pareek S.L., Prasad P.V.V., Varshney R.K. (2024). Maize and Heat Stress: Physiological, Genetic, and Molecular Insights. Plant Genome.

[2] Parankusam S., Bhatnagar-Mathur P., Sharma K.K. (2017). Heat Responsive Proteome Changes Reveal Molecular Mechanisms Underlying Heat Tolerance in Chickpea. Environ. Exp. Bot..

[3] Sharma N., Thakur M., Suryakumar P., Mukherjee P., Raza A., Prakash C.S., Anand A. (2022). ‘Breathing Out’ Under Heat Stress—Respiratory Control of Crop Yield Under High Temperature. Agronomy.

[4] Medina E., Kim S.-H., Yun M., Choi W.-G. (2021). Recapitulation of the Function and Role of ROS Generated in Response to Heat Stress in Plants. Plants.

[5] Ikeda M., Mitsuda N., Ohme-Takagi M. (2011). *Arabidopsis* HsfB1 and HsfB2b Act as Repressors of the Expression of Heat-Inducible Hsfs But Positively Regulate the Acquired Thermotolerance. Plant Physiol..

[6] Huang Y.C., Niu C.Y., Yang C.R., Jinn T.L. (2016). The Heat-Stress Factor HSFA6b Connects ABA Signaling and ABA-Mediated Heat Responses. Plant Physiol..

[7] Liu H.C., Liao H.T., Charng Y.Y. (2011). The Role of Class A1 Heat Shock Factors (HSFA1s) in Response to Heat and Other Stresses in *Arabidopsis*. Plant Cell Environ..

[8] Zhang J., Li X.M., Lin H.X., Chong K. (2019). Crop Improvement Through Temperature Resilience. Annu. Rev. Plant Biol..

[9] Rivero R.M., Mittler R., Blumwald E., Zandalinas S.I. (2022). Developing Climate-Resilient Crops: Improving Plant Tolerance to Stress Combination. Plant J..

[10] Bae H., Herman E., Bailey B., Bae H.J., Sicher R. (2005). Exogenous Trehalose Alters *Arabidopsis* Transcripts Involved in Cell Wall Modification, Abiotic Stress, Nitrogen Metabolism, and Plant Defense. Physiol. Plant..

[11] Krasensky J., Jonak C. (2012). Drought, Salt, and Temperature Stress-Induced Metabolic Rearrangements and Regulatory Networks. J. Exp. Bot..

[12] Asthir B. (2015). Mechanisms of Heat Tolerance in Crop Plants. Biol. Plant..

[13] Hassan M.U., Nawaz M., Shah A.N., Raza A., Barbanti L., Skalicky M., Hashem M., Brestic M., Pandey S., Alamri S. (2023). Trehalose: A Key Player in Plant Growth Regulation and Tolerance to Abiotic Stresses. J. Plant Growth Regul..

[14] Luo Y., Xie Y., Li W., Wei M., Dai T., Li Z., Wang B. (2021). Physiological and Transcriptomic Analyses Reveal Exogenous Trehalose Is Involved in the Responses of Wheat Roots to High Temperature Stress. Plants.

[15] Raza A., Bhardwaj S., Rahman M.A., García-Caparrós P., Habib M., Saeed F., Charagh S., Foyer C.H., Siddique K.H.M., Varshney R.K. (2024). Trehalose: A Sugar Molecule Involved in Temperature Stress Management in Plants. Crop J..

[16] Zhao D.Q., Li T.T., Hao Z.J., Cheng M.L., Tao J. (2019). Exogenous Trehalose Confers High Temperature Stress Tolerance to Herbaceous Peony by Enhancing Antioxidant Systems, Activating Photosynthesis, and Protecting Cell Structure. Cell Stress Chaperones.

[17] Luo Y., Li F., Wang G.P., Yang X.H., Wang W. (2010). Exogenously-Supplied Trehalose Protects Thylakoid Membranes of Winter Wheat from Heat-Induced Damage. Biol. Plant..

[18] Luo Y., Wang W., Fan Y.Z., Gao Y.M., Wang D. (2018). Exogenously-Supplied Trehalose Provides Better Protection for D1 Protein in Winter Wheat Under Heat Stress. Russ. J. Plant Physiol..

[19] Khan N., Bano A., Ali S., Babar M.A. (2020). Crosstalk Amongst Phytohormones from Planta and PGPR Under Biotic and Abiotic Stresses. Plant Growth Regul..

[20] Onwe R.O., Onwosi C.O., Ezugworie F.N., Ekwealor C.C., Okonkwo C.C. (2022). Microbial Trehalose Boosts the Ecological Fitness of Biocontrol Agents, the Viability of Probiotics During Long-Term Storage and Plants Tolerance to Environmental-Driven Abiotic Stress. Sci. Total Environ..

[21] Kumar S., Kaushal N., Nayyar H., Gaur P. (2012). Abscisic Acid Induces Heat Tolerance in Chickpea (*Cicer arietinum* L.) Seedlings by Facilitated Accumulation of Osmoprotectants. Acta Physiol. Plant..

[22] Lunn J.E., Delorge I., Figueroa C.M., Van Dijck P., Stitt M. (2014). Trehalose Metabolism in Plants. Plant J..

[23] Morgutti S., Negrini N., Pucciariello C., Sacchi G.A. (2019). Role of Trehalose and Regulation of Its Levels as a Signal Molecule in Abiotic Stresses in Plants. Plant Signal. Behav..

[24] Miranda J.A., Avonce N., Suárez R., Thevelein J.M., Van Dijck P., Iturriaga G. (2007). A Bifunctional TPS-TPP Enzyme from Yeast Confers Tolerance to Multiple and Extreme Abiotic-Stress Conditions in Transgenic *Arabidopsis*. Planta.

[25] Suárez R., Calderón C., Iturriaga G. (2009). Enhanced Tolerance to Multiple Abiotic Stresses in Transgenic Alfalfa Accumulating Trehalose. Crop Sci..

[26] Lyu J.I., Park J.H., Kim J.K., Bae C.H., Jeong W.J., Min S.R., Liu J.R. (2018). Enhanced Tolerance to Heat Stress in Transgenic Tomato Seeds and Seedlings Overexpressing a Trehalose-6-Phosphate Synthase/Phosphatase Fusion Gene. Plant Biotechnol. Rep..

[27] Tang C., Zhai Z., Zhong Y., Chen P., Zhou F., Zeng D., Xiang S., Song K., Guo H., Jin W. (2020). Cloning and CRISPR/Cas9-mediated targeted mutagenesis of *NtTRE* in *Nicotiana tabacum*. Life Sci. J..

[28] Zheng S., Liu C., Zhou Z., Xu L., Lai Z. (2024). Physiological and Transcriptome Analyses Reveal the Protective Effect of Exogenous Trehalose in Response to Heat Stress in Tea Plant (*Camellia sinensis*). Plants.

[29] Zheng S., Liu C., Zhou Z., Xu L., Ruan B., Chen X. (2024). Genome-Wide Identification and Characterization of Circular RNAs for Exogenous Trehalose-Mediated Heat Stress Responses in Tea Plants (*Camellia sinensis*). Front. Plant Sci..

[30] Zhang X., Chen S., Shi L., Gong D., Zhang S., Zhao Q., Zhan D., Vasseur L., Wang Y., Yu J. (2021). Haplotype-Resolved Genome Assembly Provides Insights into Evolutionary History of the Tea Plant *Camellia sinensis*. Nat. Genet..

[31] Eastmond P.J., Graham I.A. (2003). Trehalose Metabolism: A Regulatory Role for Trehalose-6-Phosphate?. Curr. Opin. Plant Biol..

[32] Zhang H., Hong Y., Huang L., Liu S., Tian L., Dai Y., Cao Z., Huang L., Li D., Song F. (2016). Virus-Induced Gene Silencing-Based Functional Analyses Revealed the Involvement of Several Putative Trehalose-6-Phosphate Synthase/Phosphatase Genes in Disease Resistance Against *Botrytis cinerea* and *Pseudomonas* syringae pv. Tomato DC3000 in Tomato. Front. Plant Sci..

[33] Zang B., Li H., Li W., Deng X.W., Wang X. (2011). Analysis of Trehalose-6-Phosphate Synthase (TPS) Gene Family Suggests the Formation of TPS Complexes in Rice. Plant Mol. Biol..

[34] Yang H.L., Liu Y.J., Wang C.L., Zeng Q.Y. (2012). Molecular Evolution of Trehalose-6-Phosphate Synthase (TPS) Gene Family in Populus, *Arabidopsis* and Rice. PLoS ONE.

[35] Yuan P., Zhou G., Yu M., Hammond J.P., Liu H., Hong D., Cai H., Ding G., Wang S., Xu F. (2024). Trehalose-6-Phosphate Synthase 8 Increases Photosynthesis and Seed Yield in *Brassica napus*. Plant J..

[36] Simola M., Hänninen A.L., Stranius S.M., Makarow M. (2000). Trehalose Is Required for Conformational Repair of Heat-Denatured Proteins in the Yeast Endoplasmic Reticulum But Not for Maintenance of Membrane Traffic Functions After Severe Heat Stress. Mol. Microbiol..

[37] Guo W., Sun Y., Chai J., Liu L., Li J., Guo R., Guo C. (2025). Genome-Wide Identification of the Trehalose-6-Phosphate Synthase Gene Family in Alfalfa (*Medicago sativa* L.) Reveals Involvement of MsTPS16 in Tolerance to Saline-Alkali Stress. Gene.

[38] Lin M., Jia R., Li J., Zhang M., Chen H., Zhang D., Zhang J., Chen X. (2018). Evolution and Expression Patterns of the Trehalose-6-Phosphate Synthase Gene Family in Drumstick Tree (*Moringa oleifera* Lam.). Planta.

[39] Zhang Z., Xiong T., Li K., Huang K., Liao C., Liu G. (2025). Evolution and Amplification of the Trehalose-6-Phosphate Synthase Gene Family in Theaceae. BMC Genom..

[40] Vandesteene L., Ramon M., Le Roy K., Van Dijck P., Rolland F. (2010). A Single Active Trehalose-6-P Synthase (TPS) and a Family of Putative Regulatory TPS-Like Proteins in *Arabidopsis*. Mol. Plant.

[41] Jiang D., Chen W., Gao J., Yang F., Zhuang C.J.B.P.R. (2019). Overexpression of the Trehalose-6-Phosphate Phosphatase OsTPP3 Increases Drought Tolerance in Rice. Plant Biotechnol. Rep..

[42] Zhang Z., Li J., Li F., Liu H., Yang W., Chong K., Xu Y. (2017). OsMAPK3 Phosphorylates OsbHLH002/OsICE1 and Inhibits Its Ubiquitination to Activate OsTPP1 and Enhances Rice Chilling Tolerance. Dev. Cell.

[43] Yuan G., Liu J., An G., Li W., Si W., Sun D., Zhu Y. (2022). Genome-Wide Identification and Characterization of the Trehalose-6-Phosphate Synthetase (TPS) Gene Family in Watermelon (*Citrullus lanatus*) and Their Transcriptional Responses to Salt Stress. Int. J. Mol. Sci..

[44] Lei M., Wu X., Huang C., Qiu Z., Wang L., Zhang R., Zhang J. (2019). Trehalose Induced by Reactive Oxygen Species Relieved the Radial Growth Defects of *Pleurotus ostreatus* Under Heat Stress. Appl. Microbiol. Biotechnol..

[45] Chen W.K., Zhang Y.Y., Lu J., Xu N.J., Sun X. (2024). Effects of Trehalose on the Physiological Parameters and Gene Expression of High-Temperature Stressed *Gracilariopsis lemaneiformis*. J. Fish. China.

[46] Raza A., Su W., Jia Z., Luo D., Zhang Y., Gao A., Hussain M.A., Mehmood S.S., Cheng Y., Lv Y. (2022). Mechanistic 48. Insights into Trehalose-Mediated Cold Stress Tolerance in Rapeseed (*Brassica napus* L.) Seedlings. Front. Plant Sci..

[47] Fu Y., Zhang Z., Liu J., Chen M., Pan R., Hu W., Guan Y., Hu J. (2020). Seed Priming with Spermidine and Trehalose Enhances Chilling Tolerance of Rice via Different Mechanisms. J. Plant Growth Regul..

[48] Chen C., Wu Y., Li J., Wang X., Zeng Z., Xu J., Liu Y., Feng J., Chen H., He Y. (2023). TBtools-II: A “One for All, All for One” Bioinformatics Platform for Biological Big-Data Mining. Mol. Plant.

[49] Kumar S., Stecher G., Suleski M., Sanderford M., Sharma S., Tamura K. (2024). MEGA12: Molecular Evolutionary Genetic Analysis Version 12 for Adaptive and Green Computing. Mol. Biol. Evol..

[50] Lescot M., Déhais P., Thijs G., Marchal K., Moreau Y., Van de Peer Y., Rouzé P., Rombauts S. (2002). PlantCARE, a Database of Plant Cis-Acting Regulatory Elements and a Portal to Tools for In Silico Analysis of Promoter Sequences. Nucleic Acids Res..

[51] Trapnell C., Williams B.A., Pertea G., Mortazavi A., Kwan G., Baren M.J.V., Salzberg S.L., Wold B.J., Pachter L. (2010). Transcript assembly and quantification by RNA-Seq reveals unannotated transcripts and isoform switching during cell differentiation. Nat. Biotechnol..

[52] Kim D., Langmead B., Salzberg S.L. (2015). HISAT: A fast spliced aligner with low memory requirements. Nat. Methods.

[53] Anders S., Huber W. (2012). Differential Expression of RNA-Seq Data at the Gene Level–The DESeq Package.

