# Supplementary figures and images for: Genome-Wide Identification, Characterization, and Expression Analysis of Trehalose Metabolism Genes in Tea Plant (Camellia sinensis) Reveals Their Roles in Response to Heat Stress
Source: Plants (Basel). 2025 Oct 29;14(21):3309. doi: 10.3390/plants14213309 (PMC12610179; doi:10.3390/plants14213309)

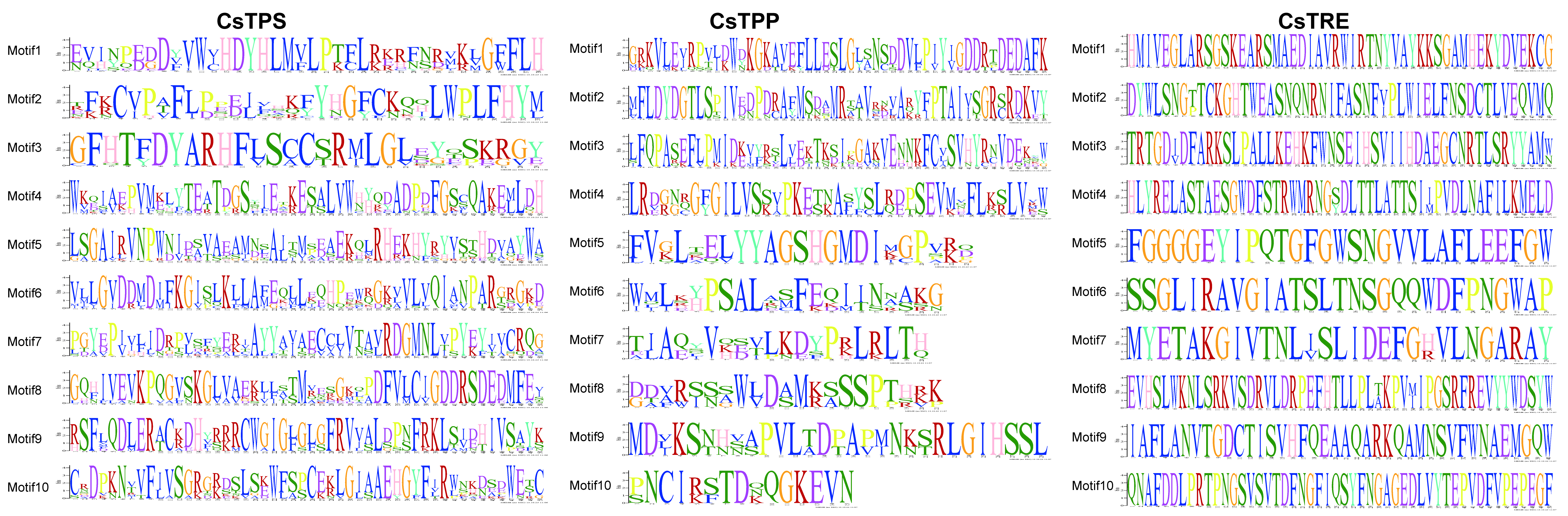

Supplement: Supplementary file 1 [file plants-14-03309-s001.zip › Figure S1.tif]

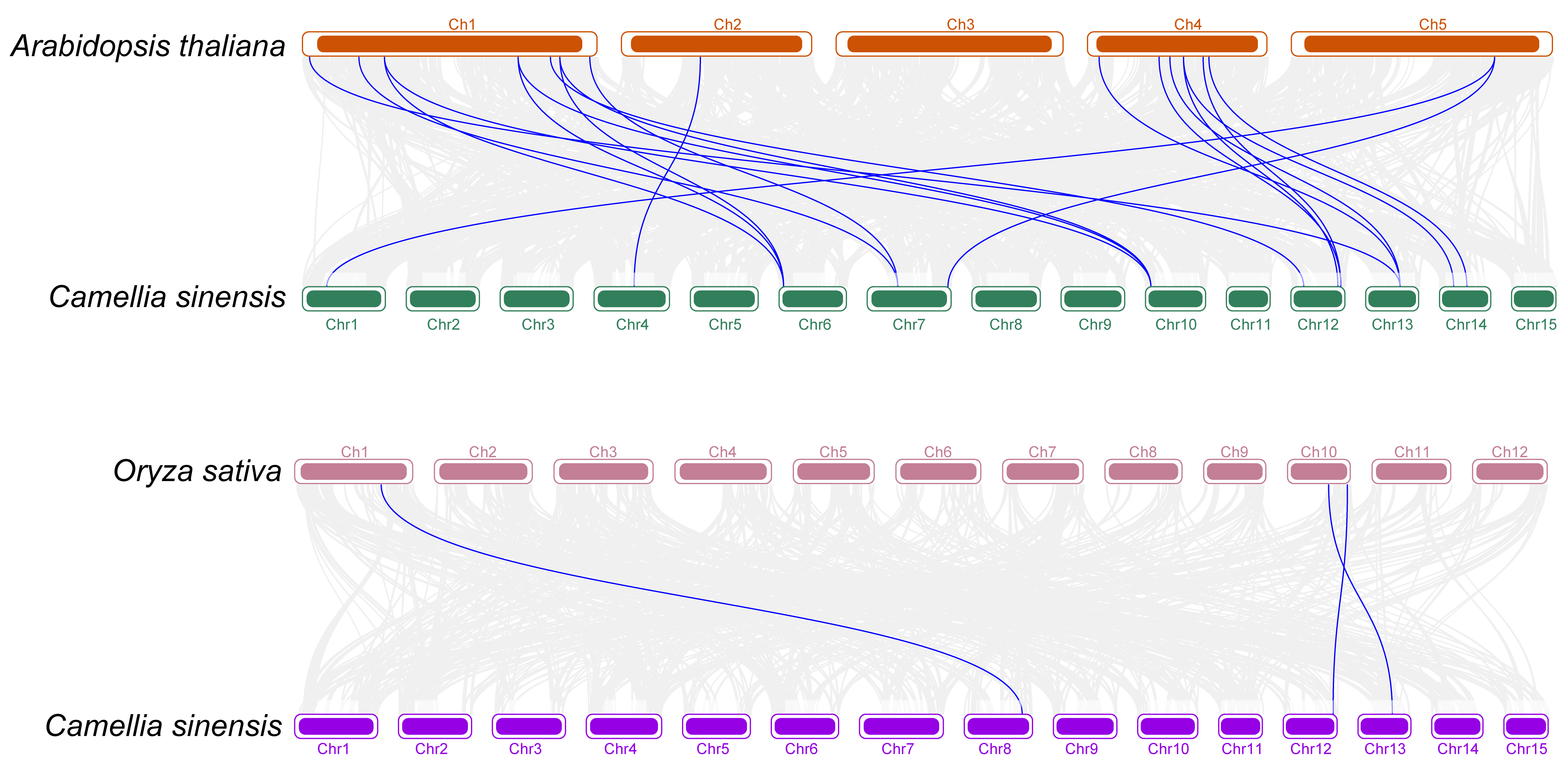

Supplement: Supplementary file 1 [file plants-14-03309-s001.zip › Figure S2.tif]
